# Node Location Privacy Protection Based on Differentially Private Grids in Industrial Wireless Sensor Networks

**DOI:** 10.3390/s18020410

**Published:** 2018-01-31

**Authors:** Jun Wang, Rongbo Zhu, Shubo Liu, Zhaohui Cai

**Affiliations:** 1 College of Computer Science, South-Central University for Nationalities, Wuhan 430074, China; jameswang@whu.edu.cn; 2 School of Computer, Wuhan University, Wuhan 430074, China; lsb_whu@126.com (S.L.); zhcai@whu.edu.cn (Z.C.)

**Keywords:** location, privacy guarantee, differential privacy, industrial wireless sensor networks

## Abstract

Wireless sensor networks (WSNs) are widely applied in industrial application with the rapid development of Industry 4.0. Combining with centralized cloud platform, the enormous computational power is provided for data analysis, such as strategy control and policy making. However, the data analysis and mining will bring the issue of privacy leakage since sensors will collect varieties of data including sensitive location information of monitored objects. Differential privacy is a novel technique that can prevent compromising single record benefits. Geospatial data can be indexed by a tree structure; however, existing differentially private release methods pay no attention to the concrete analysis about the partition granularity of data domains. Based on the overall analysis of noise error and non-uniformity error, this paper proposes a data domain partitioning model, which is more accurate to choose the grid size. A uniform grid release method is put forward based on this model. In order to further reduce the errors, similar cells are merged, and then noise is added into the merged cells. Results show that our method significantly improves the query accuracy compared with other existing methods.

## 1. Introduction

Recent years have witnessed the rapid development of the industrial wireless sensor networks (IWSNs), which have been introduced into the industry area to meet requirements of higher flexibility and market share, and IWSNs are becoming the key and fundamental technology of Industry 4.0 [1]. In the industrial domain, mobile nodes are used in industrial systems incrementally [2]. Radio modules or wireless nodes have been installed on mobile devices to raise mobility and flexibility which are ignored in traditional WSNs [3]. IWSNs generally contain more moving nodes, such as mobile products, workers and other mobile devices [4]. The centralized cloud platform collects sensor data to provide strategy control and policy making. However, the data analysis and mining will bring the issue of privacy leakage since a semi-credible cloud server is curious about sensitive location information of monitored objects [5]. To address this problem, a novel privacy protection technique called differential privacy [6] has been introduced to location privacy preservation.

**Figure 1:**
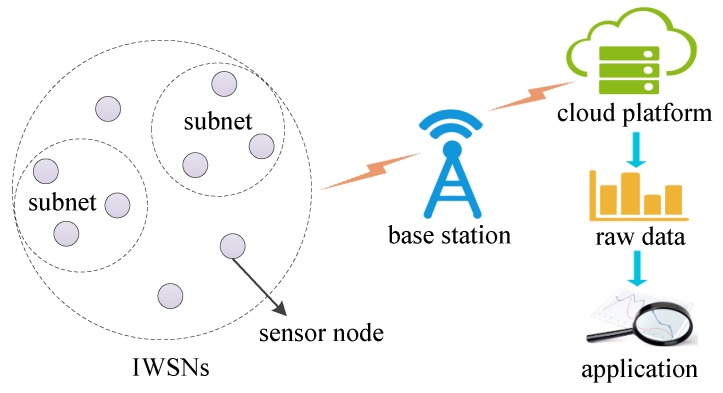
Illustration of node location data aggregation.

The Location information is called geospatial data [7]. For example, as shown in Figure 1, the location information of nodes will be collected and uploaded to the cloud. The release of static geospatial data brings great convenience to scientific research. The work in [8] indicates that it is possible to use geospatial information to forecast the spread of an infectious disease. However, data analysis also has the risk of privacy leaks. For instance, De Montjoye demonstrated that only simple date and location information of four shopping records can recognize more than 90% individuals in dataset [9]. The raw data must be sanitized before release for data analysis and mining [10].

Compared with wired networks, IWSNs have a higher possibility of privacy leaks when they integrate with cloud computing and big data technologies. Consequently, effective privacy-preserving technologies are needed in IWSNs. The work in [11] analyses existing privacy protection approaches in WSNs from data and sensor [12,13], and it investigates the approaches, such as anonymization [14], supported in large-scale industrial environments. The widespread use of geospatial data should be coupled with greater security [15], such as security controllability and strictly provable security [16]. Traditional methods based on anonymity model have risks of privacy disclosure [17,18,19]. Differential privacy is a relatively novel concept [20,21] and can hide any single record in the output by perturbing the data, which makes an adversary fail to infer the presence of a record with high probability [22,23]. How to balance between data quality and privacy level is a key problem for research [24].

In order to improve the utility of released geospatial data, a number of data decomposition methods based on tree structure were proposed [25]. In [26,27,28,29], a domain partition scheme based on quadtree or *kd*-tree is proposed to enhance the utility of released data. However, these works have not analyzed optimal partition granularity of data domains, so how to choose the appropriate tree depth or partition granularity is the key point for data decomposition strategy.

To the best of our knowledge, the work in [7] first uses a granularity partitioning scheme to create differentially private geospatial data. It assumes that the shape of query is square and the non-uniformity error is proportional to the number of location points in the cells, which fall on the border of the query shape [7]. However, the shape of data query perhaps is rectangle in actuality, i.e., the length is not equal with the width of query. Meanwhile, the larger the intersection between the border of query rectangle and cells gets, the bigger the non-uniformity error becomes. Inspired by this, we point out that the non-uniformity error is proportional to the intersection area between the border of query rectangle and cells, and a novel granularity partitioning model of data domain based on the global analysis of noise error and non-uniformity error is proposed. A bucket sort based cells merging strategy is adopted to enhance the utility of released data further, and it merges all of the similar cells contained in the data domain and differs from the scheme in [29], which just aggregates the four sub nodes of quadtree.

The contributions of this paper are as follows:

- We propose a novel granularity partitioning model of data domain, which is effective to balance the noise error and the non-uniformity error. The partition granularity is proportional to the area of data domain, privacy budget and coefficient *k*.
- We adopt a cells merging strategy based on bucket sort, which groups all the similar cells of data domain into a partition, in order to decrease the noise added to each cell. This merging strategy further raises the accuracy of query.
- We conduct evaluations using two real-world datasets to verify the effectiveness of the granularity partitioning model and the similar cells merging strategy, and results show that the proposed approach has better query accuracy and enhances the utility of released data.

The rest of this paper is organized as follows: Section 2 introduces the related work about differentially private data release, including privacy spatial decomposition (PSD) method and differentially private grids approach; Section 3 provides the preliminaries on differential privacy and problem definition; Section 4 presents the granularity partitioning model and the corresponding data release approaches; Experimental results and analysis are presented in Section 5; The conclusions and future work are finally presented in Section 6.

## 2. Related Work

The work in [30] studies the network isolation problem in group-based IWSNs, and Ref. [31] researches the dangerous area of toxic gases with WSNs. These works mostly focused on the effectiveness of network and the safety of application. As cloud and communication technologies (such as 5G and Internet) are integrated into IWSNs, more private data and information are produced. Researchers and experts are facing the serious problem of how to mine useful information from perturbed data, so data validity and privacy should be considered deeply.

In order to enhance the utility of perturbed data, PSD based on tree structure is adopted [26]. The PSD divides the geospatial dataset into m×m independent cells via horizontal and vertical lines, and gets the number of points in each cell. There are two types of error in query: one is noise error introduced by perturbation, and another is non-uniformity error generated by assuming that the location points are distributed uniformly. These two errors both affect the accuracy of query results and depend on the partition granularity *m*. The finer value of *m* implies a fewer non-uniformity error.

Based on the *kd*-tree structure, Xiao et al. [27,28] allocated half of the privacy budget to the raw data and constructed the *kd*-tree by using the sanitized data to guarantee user privacy. However, it leads to a relatively larger error [26].

Cormode et al. [26] proposed a quadtree based method Quad-opt to enhance the utility, which is called Qopt for short in this paper. Qopt splits the data domain into a complete quadtree with a predefined tree depth. Firstly, data space is divided into equal quadrants. Then, the subspace is further split into four equal pieces until tree depth reaches the predefined value *h*. Different from the existing uniform budgeting strategy, Cormode et al. proposed a novel geometric budgeting strategy, which allocates $\varepsilon_{i}$ for each level of the quadtree ($\sum_{i=0}^{h}\varepsilon_{i}=\varepsilon$). Notably, proportional factor is $2^{1/3}$ and constrained inference [32] is employed to post-process the output of query with the purpose of higher utility. However, directly adding noise into each cell will result in a larger error when the data is sparse.

Fan et al. [29] proposed to aggregate similar cells into a partition to overcome the data sparsity issue. First, each cell is pre-classified by domain knowledge, i.e., the cell is sparse or dense type. Next, a node is split into four equal quadrants until the predefined depth value *h* is reached or the node is homogeneous, i.e., all the cells within the node belong to the same type. This method needs to pre-classify the type of cells relying on the specialized knowledge, which may lead to misjudgment. Meanwhile, merging of cells just restricts in quadtree node and fails to extend to the entire data domain.

For all above methods based on PSD, the utility of perturbed data is related to tree depth *h* or partition granularity *m*, so how to choose the right value of *h* or *m* is the key point. Qardaji et al. [7] proposed a granularity partitioning model of data domain to solve this problem and presented a uniform method UG based on this model. The partition granularity is $\sqrt{N\varepsilon /c}$, where *N* is the number of data points in cell, *c* is a small constant (generally *c* = 10), and ε is the privacy budget. Qardaji et al. further proposed an adaptive grid method AG. It splits the data domain into $m_{1}\times m_{1}$ independent cells, where $m_{1}=max\left(10,0.25\times \left\lceil N\varepsilon /c\right\rceil \right)$ . The cell will be further divided into $m_{2}\times m_{2}$ independent cells if the noisy count of the cell is bigger than the given threshold value, where $m_{2}=\left\lceil 2N^{\prime }\left(1-\xi \right)\varepsilon /c\right\rceil$, and ξ is a parameter determined by user. To et al. [33] adopted the AG method to solve the spatial crowdsourcing specific requirements, and they modified the parameter *c* to decrease the system overhead, which is just beyond our research. Note that literature [7] assumes that the length and width of query rectangle are equal to each other, which may affect the utility of perturbed data.

In this paper, the proposed method Ugrid adopts a novel granularity partitioning model of data domain. There is no need to assume that the length and width of query rectangle are equal to each other, and the partition granularity is $\left\lceil \sqrt{4kHL\varepsilon /\sqrt{2}}\right\rceil$ , where *k* is the proportionality coefficient, *H* and *L* are the width and length of data domain, respectively, and ε is the privacy budget. We further introduce a merging grids release approach, which groups all of the similar cells into a partition and adds Laplace noise to each partition. The aggregation of the similar cells has improved the accuracy of data query.

## 3. Preliminaries

In this section, we formally introduce the basic concept of differential privacy and present the problem definition.

### 3.1. Differential Privacy

The formalized definition of differential privacy is as follows.

**Definition 1.**  *Differential privacy [6]: Given two neighboring datasets D and D’, which differ at most one tuple, and a randomized algorithm A:D→R. Let O be the set of all possible outputs of algorithm A, and A is said to satisfy ε-differential privacy for any subset σ⊆O if*

$$\frac{Pr[A(D)\in \sigma ]}{Pr[A\left(D^{\prime }\right)\in \sigma ]}\leq exp\left(\varepsilon \right).$$

The parameter ε is called privacy budget, which is used to control the ratio of output of algorithm *A* in neighboring datasets *D* and *D’* [25]. Smaller ε yields a stronger privacy guarantee because the output probabilities of algorithm *A* in *D* and *D’* are approximately the same, which makes the adversary fail to judge whether the tuple is present in the dataset or not. However, the smaller ε is, the lower the utility will be, as the adding noise is bigger. Detail of noise addition can be seen in the part of the Laplace mechanism. The value of ε is usually small, such as 0.1, 0.5, 1, etc. [25].

Differential privacy owns a significant composable property, which plays an important role in demonstrating whether an algorithm satisfies ε-differential privacy or not.

**Property 1.**  *Parallel composition [34]: Let random algorithm $A_{i}$ each provide $\varepsilon_{i}$-differential privacy for disjoint subsets $D_{i}$, and the sequence of $A_{i}\left(D_{i}\right)$ provides $max(\varepsilon_{i})$- differential privacy.*

**Property 2.**  *Sequential composition [34]: Let random algorithm $A_{i}$ each provide $\varepsilon_{i}$-differential privacy. Then, a sequence of $A_{i}\left(D\right)$ over the database D provides $\Sigma \varepsilon_{i}$- differential privacy.*

Laplace mechanism is a differentially private implementation scheme, which masks the real data by adding random noise following Laplace distribution to the output. The value of noise is related to privacy budget ε and global sensitivity.

**Definition 2.**  *Global sensitivity [6]: Given a function $f:D\to R^{d}$, the global sensitivity of f is defined as follows:*

$$\tau \left(f\right)=\max_{D,D^{\prime }}||f\left(D\right)-f\left(D^{\prime }\right){\left|\right|}_{1}.$$

The parameter *D* and $D^{\prime }$ are neighboring datasets; *R* is the real space; *d* is the dimension; and $||f\left(D\right)-f\left(D^{\prime }\right){\left|\right|}_{1}$ is the first-order norm distance [25]. For instance, the global sensitivity of count function is 1.

**Definition 3.**  *Laplace mechanism [35]: Given a dataset D and a function $f:D\to R^{d}$, if the adding noise follows Laplace distribution, i.e., noise∼Lap(τ(f)/ε), where location parameter is 0, scale parameter is τ(f)/ε; then, random algorithm A(D)=f(D)+noise provides ε-differential privacy.*

The exponential mechanism addresses the non-numeric case in which adding noise makes no sense. It is another method to construct differentially private algorithm over any quality function u(D,r).

**Definition 4.**  *Exponential mechanism [36]: Given a dataset D, a privacy parameter ε, a quality function u(D,r), and the global sensitivity τ(u) of u(D,r), random algorithm A provides ε-differential privacy if algorithm A chooses an outcome r from the range R with probability*

$$A\left(D,u\right)=\{r:|Pr\left[r\in R\right]\propto exp\left(\frac{\varepsilon }{2\tau (u)}u\left(D,r\right)\right)\}.$$

Let Lap(b) be a Laplace distribution, where location parameter μ=0, scale parameter is *b*, and its probability density function is p(x)=exp(−|x|/b)/2b. According to the function p(x), the bigger *b* gets, the bigger perturbed noise becomes.

If noise∼Lap(b), let σ(x) denotes standard deviation, D(x) denote variance, $\sigma \left(x\right)=\sqrt{D(x)}$, $D\left(x\right)=2b^{2}$, and b=τ(f)/ε; then, $D\left(x\right)=2b^{2}=2\tau {\left(f\right)}^{2}/\varepsilon^{2}$, $\sigma \left(x\right)=\sqrt{D(x)}=\sqrt{2\tau {\left(f\right)}^{2}/\varepsilon^{2}}=\sqrt{2}\tau \left(f\right)/\varepsilon$ [25].

### 3.2. Problem Definition

**Figure 2:**
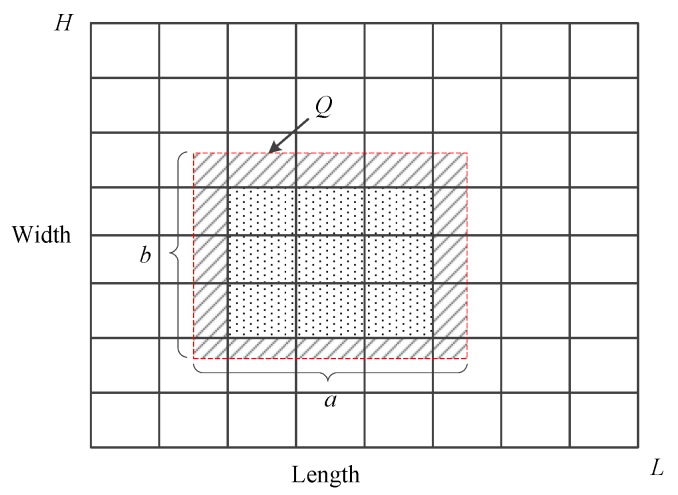
Example of data query.

Let *L* and *H* be the domain length and width of geospatial dataset *D* of monitored objects in IWSNs; *a* and *b* are the length and width of data query *Q* as shown in Figure 2. Splitting the data domain into m×m cells $\left\{c_{1},c_{2},\ldots ,c_{i},\ldots ,c_{m\times m}\right\}$, point count $x_{i}$ of cell $c_{i}$ is perturbed by random noise following Laplace distribution. Then, the perturbed differentially privacy dataset $\tilde{D}=\left\{{\tilde{x}}_{1},{\tilde{x}}_{2},\ldots ,{\tilde{x}}_{i},\ldots ,{\tilde{x}}_{m\times m}\right\}$ is released.

Let $e_{n}$ be the noise error introduced by the addition noise, and let $e_{u}$ be the non-uniformity error caused by the assumption of uniform distribution. As shown in Figure 2, there is an intersection between cells and query rectangle *Q*, and some cells are partly contained in it, such as cells filled with oblique lines. For these cells, we calculate the number of data points in the intersected part based on the uniformity assumption. For instance, let $I_{i}$ be the intersected part between cell $c_{i}$ and query *Q*, and let $x_{i}^{\prime }$ be the estimated count of data points in $I_{i}$ . Then, $x_{i}^{\prime }=x_{i}\times area\left(I_{i}\right)/area\left(c_{i}\right)$, where $x_{i}$ is the count of data points in $c_{i}$, $area(I_{i})$ is the area of $I_{i}$, and $area(c_{i})$ is the area of $c_{i}$. We conclude that $x_{i}^{\prime }=x_{i}$ if $area\left(I_{i}\right)=area\left(c_{i}\right)$, $x_{i}^{\prime }\neq x_{i}$ if not.

Intuitively, a bigger value of partition granularity implies a smaller non-uniformity error and a larger noise error when splitting the data domain. In contrast, a smaller value of partition granularity means a smaller noise error and a larger non-uniformity error because the value of partition granularity *m* is the key. Qardaji et al. inferred the value of *m* based on the assumption a=b [7], which is needless in this paper.

From the above analysis, given a geospatial dataset *D* and privacy budget ε, how to choose an optimal partition granularity *m* to minimize the error of query *Q* is the research question in this paper. The formalized definition is defined as follows:

$$\min_{m}\left(e_{n}+e_{u}\right).$$

Table 1 is a summary of the primary symbols used in this paper.

## 4. Sanitized Data Release Based on Grid Partition

This section mainly states the sanitized data release approach Ugrid and Mgrid. In particular, the granularity partitioning model of data domain is first presented; then, Ugrid and Mgrid based on this model are presented.

### 4.1. Uniform Grid Release Approach

#### 4.1.1. Granularity Partitioning Model of Data Domain

We give the optimal value of partition granularity *m* based on the overall analysis of perturbed data’s noise error and non-uniformity error. Details of derivation are as follows.

As illustrated in Figure 2, let cells that are filled with oblique lines be *I*, the non-uniformity error is 0 when the area of *I* is 0; now, cells in *Q* are completely contained in it. The non-uniformity error becomes bigger with the area of *I* increasing when cells in *Q* are not contained in it. Motivated by this, we propose that the relative error β based on the uniformity assumption is proportional to the area α of *I*. Let *k* be the proportionality coefficient between β and α, $\beta_{i}$ and $\alpha_{i}$ are the *i*th sampling values; let $\tilde{\beta }$ and $\tilde{\alpha }$ be the mean value of relative error and area of *I*, and we can infer the value of *k* through linear-regression analysis, and the value of *k* can be calculated by least square estimation; the formula is as follows:

k=∑(αi−α¯)(βi−β¯)∑(αi−α¯)(βi−β¯)∑(αi−α¯)2∑(αi−α¯)2.

For instance, we have that relative error satisfies β=0.1291α+const through linear-regression analysis in checkin dataset, where *k* = 0.1291. Notably, the relative error is 0 when the area of *I* is 0. We finally set *k* = 0.1314 after using the coordinate origin (0, 0) to correct the value of *k*. Details of checkin dataset can be seen in Section 5.

The bigger the area of *I* gets, the bigger the non-uniformity error becomes. The value of the non-uniformity error $e_{u}$ reaches a maximum in theory, and the area of *I* is the region of cells that intersected with the border of the query *Q*. The value of $e_{u}$ is k(2aH+2bL)/m.

The analysis of non-uniformity error: the number of cells that intersected with the border of *Q* is defined as Num=2am/L+2bm/H, where 2am/L is the number of cells that intersected with the top and bottom borders of *Q*, and 2bm/H is the number of cells intersected with the left and right borders of *Q*. The area of each cell is $LH/m^{2}$, where *LH* is the total area of data domain and $m^{2}$ is the total number of cells. Then, we deduce the total area of the cells that intersected with the border of *Q* is $Num\times LH/m^{2}=\left(2am/L+2bm/H\right)\times LH/m^{2}$, and non-uniformity error that is proportional to the total area is $k\left(2am/L+2bm/H\right)\times LH/m^{2}$.

The noise error is caused by the added Laplace noise and is affected by the value of partition granularity *m*. The value of $e_{n}$ is $\sqrt{2r}m/\varepsilon$, and r=ab/LH.

The analysis of noise error: the added random noise follows Laplace distribution and has a standard deviation $\sqrt{2}/\varepsilon$. The number of cells included in the query *Q* is $Num^{\prime }=\left(ab/LH\right)m^{2}$, where ab/LH is the ratio of the area of query *Q* to the area of data domain and $m^{2}$ is the total number of cells. Thus, the standard deviation of the total noise error is $\sqrt{2Num^{\prime }}/\varepsilon =\sqrt{2\left(ab/LH\right)m^{2}}/\varepsilon =\sqrt{2r}m/\varepsilon$, and r=ab/LH.

**Lemma** **1.** The total error of data query is at a minimum when $m=\lceil \sqrt{4kHL\varepsilon /\sqrt{2}}\rceil$, where k is the proportional coefficient, H and L are the domain width and length of dataset D, and ε is the privacy budget.

**Proof.**  The total error is the sum of non-uniformity error $e_{u}$ and noise error $e_{n}$, where $e_{u}=k\left(2am/L+2bm/H\right)\times LH/m^{2}=k\left(2aH+2bL\right)/m\geq 2k\sqrt{4aH\times bL}/m=4k\sqrt{HL}\times$$\sqrt{rHL}/m=4kHL\sqrt{r}/m,e_{n}=\sqrt{2r}m/\varepsilon$, according to the above analysis. To minimize the total error, according to $4kHL\sqrt{r}/m=\sqrt{2r}m/\varepsilon$, we deduce that $m=\sqrt{4kHL\varepsilon /\sqrt{2}}$, and round it up to a whole number; then, $m=\lceil \sqrt{4kHL\varepsilon /\sqrt{2}}\rceil$. ☐

#### 4.1.2. Ugrid Method

We propose a uniform grid release approach based on this granularity partitioning model. First of all, Ugrid splits the data domain into m×m independent cells according to the value of partition granularity *m*, where $m=\lceil \sqrt{4kHL\varepsilon /\sqrt{2}}\rceil$. Next, calculate the count $x_{i}$ of data points in each cell $c_{i}$ through a traversal of *D* and getting the point counts of all cells $\left\{x_{1},x_{2},\ldots ,x_{i},\ldots ,x_{m\times m}\right\}$. Then, obtain the noised count ${\tilde{x}}_{i}=x_{i}+Lap\left(1/\varepsilon \right)$ by adding Laplace noise Lap(1/ε). Finally, share the sanitized dataset $\tilde{D}=\left\{{\tilde{x}}_{1},{\tilde{x}}_{2},\ldots ,{\tilde{x}}_{i},\ldots ,{\tilde{x}}_{m\times m}\right\}$ for query services.

The pseudo code description of Ugrid is presented in Algorithm 1. Steps 1∼5 of Ugrid conduct domain division in geospatial dataset *D*; step 6 sets point count $x_{i}=\left|c_{i}\right|$; in steps 7∼9, the Laplace noise is added into each count $x_{i}$ of $c_{i}$; and the last step generates the differentially privacy sanitized dataset $\tilde{D}$.

**Algorithm 1** Ugrid**Input:** geospatial dataset *D*, privacy budget ε, partition granularity *m***Output:** sanitized dataset $\tilde{D}=\left\{{\tilde{x}}_{1},{\tilde{x}}_{2},\ldots ,{\tilde{x}}_{i},\ldots ,{\tilde{x}}_{m\times m}\right\}$  1:**for** (o=1;o≤D.size();o++) **do**  2:   **if** $\left(point_{o}\in c_{i}\right)$ **then**  3:       add $point_{o}$ to cell $c_{i}$  4:   **end if**  5:**end for**  6:set point count $x_{i}=\left|c_{i}\right|$  7:**for** (i=1;i≤m×m;i++) **do**  8:   noisy count ${\tilde{x}}_{i}=x_{i}+Lap\left(1/\varepsilon \right)$  9:**end for**10:sanitized dataset $\tilde{D}=\left\{{\tilde{x}}_{1},{\tilde{x}}_{2},\ldots ,{\tilde{x}}_{i},\ldots ,{\tilde{x}}_{m\times m}\right\}$

**Theorem** **1.** Algorithm Ugrid satisfies ε-differential privacy.

**Proof.**  According to Property 1 parallel composition, Laplace noise Lap(1/ε) is added into m×m independent cells; then, Ugrid provides $max(\varepsilon_{i})$ differential privacy. As $\varepsilon_{i}=\varepsilon$, Ugrid satisfies ε-differential privacy. ☐

**Theorem** **2.** Given geospatial dataset D and partition granularity m, the time complexity of Ugrid is $O(|D|+m^{2})$.

**Proof.**  In steps 1–5 of algorithm, dataset *D* has |D| data points, and the cost is |D|. In steps 7–9, there are $m^{2}$ cells, and the cost is $m^{2}$. Therefore, the total cost is $O(|D|+m^{2})$. ☐

To balance the noise error and non-uniformity error, Ugrid sets $m=\lceil \sqrt{4kHL\varepsilon /\sqrt{2}}\rceil$ to minimize the total error. However, the output of query may contain a large mass of noise when some cells are extremely sparse. For example, let real count $x_{i}$ of cell $c_{i}$ be 1, the added noise is 20, and then the noise error is 20, which remains with little valuable information in it. In order to decrease data query error and enhance the utility of perturbed data, we further introduce a merging grids release approach Mgrid. It aggregates all similar cells into a partition employing the bucket sort based cell merging strategy, which reduces the noise error by adding noise into each partition.

### 4.2. Merging Grids Release Approach

#### 4.2.1. Merging of Similar Cells

The formalized definition of similar cells merging is defined as follows:

**Definition 5.**  *Similar cells merging: Given the point count of all cells $\{x_{1},x_{2},\ldots ,x_{i},$$\ldots ,x_{m\times m}\}$, they are similar if the count of these cells:*

$$|x_{i}.hash-x_{j}.hash|<c,i,j\in N^{*}.$$

The parameter *c* is a constant, and corresponding cells will be merged into a partition if the difference between every two hash value of these cells is smaller than the given threshold *c*. BKDRHash maps the binary string(3-bits are a group) of $x_{i}$ to $x_{i}.hash$.

To find the similar cells, clustering algorithm affinity propagation [37] has the advantage of not needing to specify the cluster “number”. However, the algorithm is more complex, and the time complexity is $O(m^{4}\log m^{2})$. To enhance the efficiency, the bucket sort based similar cells merging is adopted. Given mapping function $f\left(x_{i}\right)=x_{i}.hash/c,i\in \left[1,m^{2}\right]$, *c* is a small value, and is related to the BKDRHash. Mapping each cell with count $x_{i}$ to the corresponding bucket, the cells in each bucket just are similar cells. Bucket sort based similar cells merging traverses all cells only just once, and its time complexity is $O(m^{2})$.

**Figure 3:**
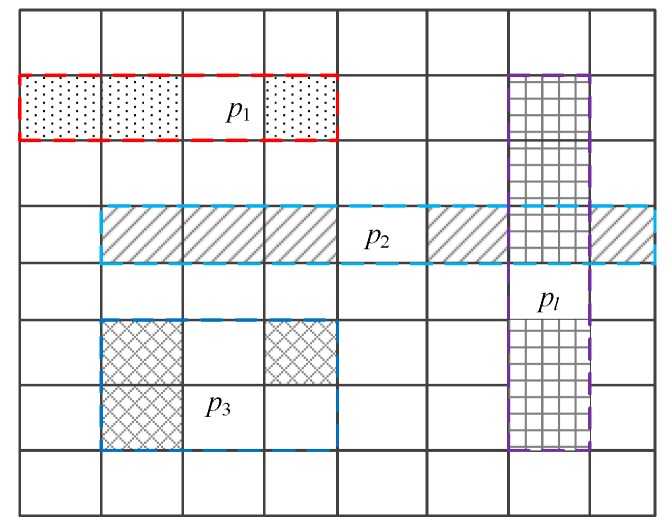
Example of merging cells.

After the merging of similar cells, we get the partition dataset $\left\{p_{1},p_{2},\ldots ,p_{l},\ldots ,p_{L}\right\}$. As shown in Figure 3, cells filled with point symbols, oblique lines, diagonal grids, horizontal and vertical grids are merged into the partitions $p_{1}$, $p_{2}$, $p_{3}$, $p_{l}$, respectively. The random noise Lap(1/ε) is added to each partition $p_{l}$; then, the noise added to each cell contained in partition is decreased. Note that BKDRHash is used in the process of similar cells merging to protect data privacy of the real count of all cells.

#### 4.2.2. Mgrid Method

We further propose the merging grids release approach based on the granularity partitioning model and the similar cells merging strategy. Similar to the method Ugrid, firstly, Mgrid splits the dataset into m×m cells $\{c_{1},c_{2},\ldots ,c_{i},\ldots ,$$c_{m\times m}\}$ based on the granularity *m*. Secondly, it traverses *D* and calculates the hash value $x_{i}.hash$ of $x_{i},i\in \left[1,m^{2}\right]$ based on the BKDRHash. Thirdly, similar cells are selected according to the mapping function *f*. Furthermore, Laplace noise Lap(1/ε) is added to each partition $p_{l},l\in \left[1,L\right]$, and noisy count ${\tilde{x}}_{i}$ of each cell $c_{i}$ in $p_{l}$ is defined as ${\tilde{x}}_{i}=\left(\right|p_{l}\left|+Lap\left(1/\varepsilon \right)\right)/p_{l}.size\left(\right)$, where $|p_{l}|$ is the count of data points located in $p_{l}$. Finally, the sanitized dataset $\tilde{D}=\left\{{\tilde{x}}_{1},{\tilde{x}}_{2},\ldots ,{\tilde{x}}_{l},\ldots ,{\tilde{x}}_{L}\right\}$ is released for query services.

Algorithm 2 states the pseudo code of Mgrid. Steps 1–5 of the Mgrid conduct the cell division in geospatial dataset *D*; in steps 7–9, each count $x_{i}$ is mapped to corresponding bucket according to the mapping function $f\left(x_{i}\right)=x_{i}.hash/c$; step 10 gets the partition dataset $\left\{p_{1},p_{2},\ldots ,p_{l},\ldots ,p_{L}\right\}$ after the merging of similar cells; in steps 11–13, the Laplace noise is added into each partition $p_{l}$; the final step generates the sanitized differentially privacy dataset $\tilde{D}$.

**Algorithm 2** Mgrid**Input:** geospatial dataset *D*, privacy budget ε, partition granularity *m*, threshold *c***Output:** sanitized dataset $\tilde{D}=\left\{{\tilde{x}}_{1},{\tilde{x}}_{2},\ldots ,{\tilde{x}}_{l},\ldots ,{\tilde{x}}_{L}\right\}$  1:**for** (o=1;o≤D.size();o++) **do**
  2:   **if** $\left(point_{o}\in c_{i}\right)$ **then**  3:       add $point_{o}$ to cell $c_{i}$  4:   **end if**  5:**end for**  6:set point count $x_{i}=\left|c_{i}\right|$  7:**for** (i=1;i≤m×m;i++) **do**  8:   select similar cells by mapping function $f\left(x_{i}\right)=x_{i}.hash/c$  9:**end for**10:set partition dataset is $\left\{p_{1},p_{2},\ldots ,p_{l},\ldots ,p_{L}\right\}$, where $p_{l}$ = {similar cells}11:**for** (l=1;l≤L;l++) **do**12:   noisy count ${\tilde{x}}_{i}=\left|p_{l}\right|+Lap\left(1/\varepsilon \right)$13:**end for**14:sanitized dataset $\tilde{D}=\left\{{\tilde{x}}_{1},{\tilde{x}}_{2},\ldots ,{\tilde{x}}_{l},\ldots ,{\tilde{x}}_{L}\right\}$


Mgrid also satisfies ε-differential privacy, and the proof is similar to Ugrid’s. Note that the Laplace noise is added to each partition, bucket sort does not consume the privacy budget, and the Laplace mechanism consumes ε.

**Theorem** **3.** Data query error is less than or equal to the value before the merging of similar cells.

**Proof.**  Given the geospatial dataset, before the merging of similar cells, the noise error $e_{n}$ of query *Q* is $\sum_{i=1}^{\sqrt{r}m}\sqrt{2}/\varepsilon$, and non-uniformity error $e_{u}$ is $k\left(2am/L+2bm/H\right)\times LH/m^{2}$=k(2aH+2aL)/m, the data query error is then $error=e_{n}+e_{u}=\sum_{i=1}^{\sqrt{r}m}\sqrt{2}/\varepsilon +k\left(2aH+2bL\right)/m$.

After the merging of similar cells, the noise error of query *Q* is $e_{n}^{\prime }=\sum_{i=1}^{\sqrt{r}m}\sqrt{2}/\varepsilon p_{l}.size\left(\right)$, $l\in \left[1,L\right],p_{l}.size\left(\right)\geq 1$, and non-uniformity error is $e_{u}^{\prime }=k\left(2am/L+2bm/H\right)\times LH/m^{2}=k\left(2aH+2aL\right)/m$; then, the data query error is $error^{\prime }=e_{n}^{\prime }+e_{u}^{\prime }=\sum_{i=1}^{\sqrt{r}m}\sqrt{2}/$$\varepsilon p_{l}.size\left(\right)+k\left(2aH+2bL\right)/m$.

The difference between $error^{\prime }$ and error is $(\sum_{i=1}^{\sqrt{r}m}\sqrt{2}/$$\varepsilon )\left(1/p_{l}.size\left(\right)-1\right)\leq 0$, so we deduce that data query error is less than or equal to the value before the merging of similar cells. ☐

**Theorem** **4.** Given geospatial dataset D and partition granularity m, then the time complexity of Mgrid is $O(|D|+m^{2}+L)$.

**Proof.**  In steps 1–5 of algorithm, dataset *D* has |D| data points, and the cost is |D|. There are $m^{2}$ cells in steps 7–9, and the cost is $m^{2}$. In steps 11–13, the number of all partitions is *L*. Therefore, the total cost is $O(|D|+m^{2}+L)$. ☐

## 5. Experimental Results and Analysis

In this section, we begin with the introducing of the metric standard of perturbed data. Then, we present the experimental datasets used in this paper. Finally, detailed analysis of experimental results is presented.

### 5.1. Utility Metric

There is no specific utility metric standard in existing literature for differential privacy, and the existing methods usually adopt variance [26,38], relative error [7,26,29], absolute error [7], etc. to evaluate the utility of sanitized data. In this paper, we adopt relative error and absolute error to estimate the utility of sanitized data. Given a query *Q*, let Q(D) denote the real query result, let $Q(\tilde{D})$ denote the noisy query result, and then the relative error is defined as:

Error(Q)=Q(D)−Q(D˜)Q(D)−Q(D˜)max{Q(D),DD4646}max{Q(D),DD4646}.

The parameter |D| is the count of nodes contained in dataset *D*, and the divisor is $\left|D\right|/4^{6}$ when Q(D)=0, which avoids dividing by zero. The smaller the relative error is, the more accurate the query will be. Meanwhile, in order to intuitively observe the value of added noise, we also present the comparison results of absolute error.

### 5.2. Experimental Datasets

**Figure 4:**
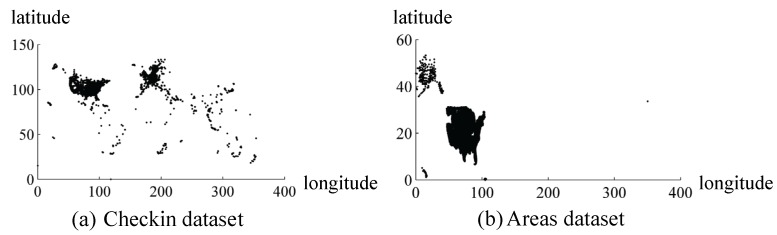
Illustration of datasets.

In Figure 4, the shapes of datasets are presented by plotting the point of monitored object directly, and the coordinate of base point starts from (0, 0) for intuition and convenience.

The first dataset is named as Checkin [39] obtained from a location-based social networking website Gowalla as illustrated in Figure 4a. It mainly includes the location point, check-in-time, and location id with about 6,442,890 records and only part of the location point information is used. The length and width of the data query *Q* increase by 10 in this dataset.

The second dataset Areas [40] is obtained from the U.S. Census Bureau as illustrated in Figure 4b. Dataset Areas is the legislative areas national geodatabase, and we only employ the location point data. The length of *Q* increases by 3 and the width increases by 2 in this dataset.

The query error is verified under different privacy budgets, such as 0.1, 0.5 and 1. Table 2 presents the parameters information about these datasets.

We randomly generate 500 data queries for each query size and finally calculate the mean value. The experiments were conducted on Intel i5 CPU (santa clara, CA, USA) with 3 GB RAM, the programming platform is Eclipse3.5 (Eclipse Foundation, Inc., Ottawa, Canada), and the programming language is JAVA.

### 5.3. Experimental Results

This subsection presents the experimental results of UG [7], AG [7], Ugrid, Mgrid, and Qopt under different query *Q*.

**Figure 5:**
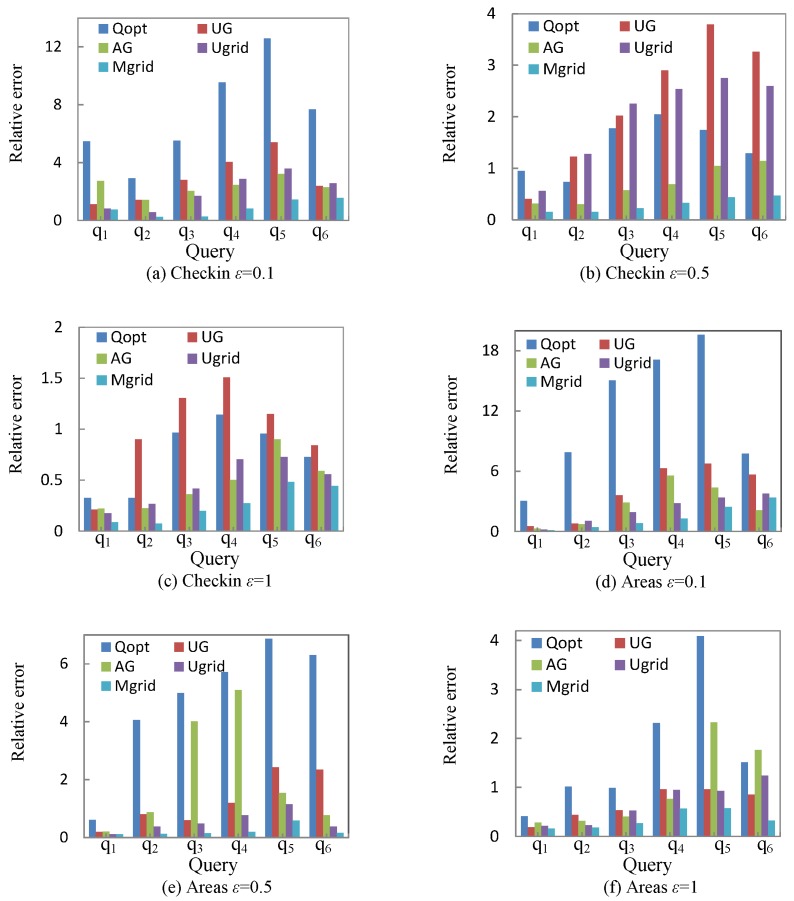
Relative error of query.

Figure 5 shows the relative error in different queries. Compared with other methods, Mgrid outweighs other methods on a whole. The relative error of method Ugrid is comparatively large as shown in Figure 5b, and we believe this kind of situation is caused by excessive sparse cells contained in query *Q*. Mgrid further enhances the query accuracy by the similar cells merging strategy, which merges the similar cells into a partition to decrease the added noise to each cell. Experimental results verify the effectiveness of the granularity partitioning model and the similar cells merging strategy.

Note that the experimental results of UG outperform the results of AG in Figure 5e. This situation may be caused by the privacy budget allocation and the sparsity of dataset. AG allocates half of privacy budget to the first-level cells and the other half of the budget to the second-level cells; different allocation strategy will have an impact on the query results.

**Figure 6:**
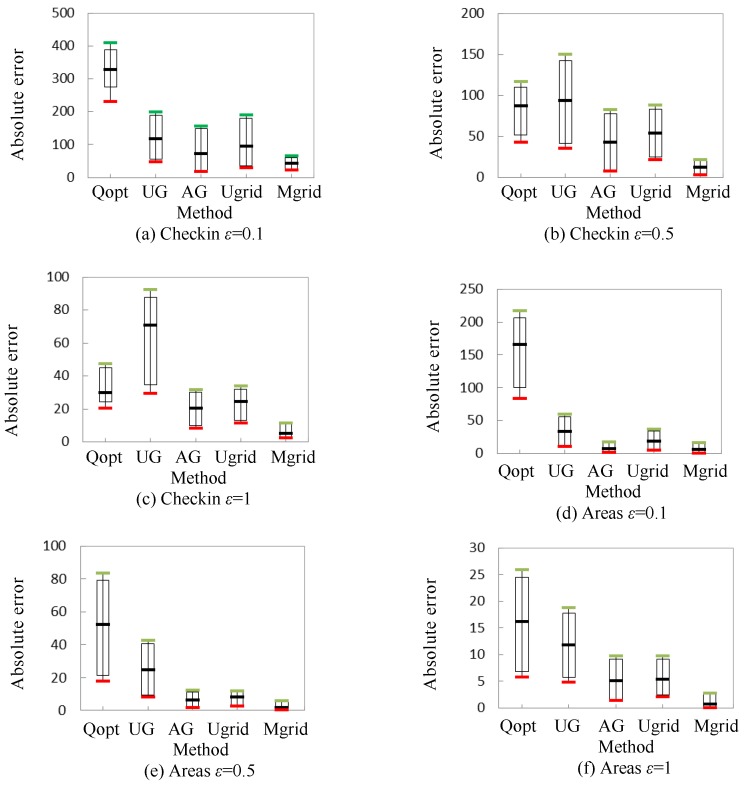
Absolute error of query.

Figure 6 shows the profile of absolute error, which is displayed by candlestick chart, in different queries. Note that the top horizontal line of the candlestick is the maximum value, the bottom horizontal line of the candlestick is the minimum value, and the middle horizontal line of the candlestick is the arithmetic mean. The top of the box is 95% of the maximum value, and the bottom of the box is 120% of the minimum value.

As shown in Figure 6, just considering the absolute error of the added noise, we also deduce that Mgrid outperforms the four other methods. Note that the results of Qopt in dataset Areas are getting worse, which shows that a granularity partition based method works well when dealing with the sparse data.

## 6. Conclusions

The location information of monitored objects is an important privacy attribute in IWSNs, which has become a significant research direction. To protect node location privacy and enhance the utility of perturbed data, we propose a novel granularity partitioning model based on the overall analysis of two types of errors. This model considers that the shape of query *Q* is a rectangle, which is closer to the requirement of actual queries. Ugrid and Mgrid are proposed based on this model and are validated through two real world datasets. Experimental results show that Mgrid has a good utility and query accuracy.

As for future work, we will continue to improve the granularity partitioning model and make the model to be in better accordance with the self-characteristics of datasets. For instance, using area and point count two factors to construct the model. In addition, we will explore the distributed application for geospatial datasets—for example, multiserver differentially private data release.

## Figures and Tables

**Table 1:** Symbols.

Symbol	Description
*D*	two-dimensional geospatial dataset
$\tilde{D}$	sanitized dataset
$c_{i}$	cell
$x_{i}$	count of data points in cell $c_{i}$
*k*	proportionality coefficient
*Q*	data query
$I_{i}$	intersection between query *Q* and cell $c_{i}$
${\tilde{x}}_{i}$	noisy count of cell $c_{i}$
*L*	domain length of *D*
*H*	domain width of *D*
*a*	length of *Q*
*b*	width of *Q*

**Table 2:** Parameter information about datasets.

Dataset	Num of Points	Domain Size	Query Size
Checkin	625,123	354 × 133	$q_{i}=10\left(i+1\right)\times 10\left(i+1\right),i\in \left[1,6\right]$
Areas	179,371	351 × 54	$q_{i}=3\left(i+5\right)\times 2\left(i+5\right),i\in \left[1,6\right]$

## Abbreviations

The following abbreviations are used in this paper:

IWSNs	Industrial Wireless Sensor Networks
WSNs	Wireless Sensor Networks
PSD	Privacy Spatial Decomposition
